# The contribution of office work to sedentary behaviour associated risk

**DOI:** 10.1186/1471-2458-13-296

**Published:** 2013-04-04

**Authors:** Sharon Parry, Leon Straker

**Affiliations:** 1 School of Physiotherapy, Curtin University, Perth Western Australia, GPO Box U1987, Perth, WA 6845, Australia

## Abstract

**Background:**

Sedentary time has been found to be independently associated with poor health and mortality. Further, a greater proportion of the workforce is now employed in low activity occupations such as office work. To date, there is no research that specifically examines the contribution of sedentary work to overall sedentary exposure and thus risk. The purpose of the study was to determine the total exposure and exposure pattern for sedentary time, light activity and moderate/vigorous physical activity (MVPA) of office workers during work and non-work time.

**Methods:**

50 office workers from Perth, Australia wore an Actical (Phillips, Respironics) accelerometer during waking hours for 7 days (in 2008–2009). Participants recorded wear time, waking hours, work hours and daily activities in an activity diary. Time in activity levels (as percentage of wear time) during work and non-work time were analysed using paired t-tests and Pearson’s correlations.

**Results:**

Sedentary time accounted for 81.8% of work hours (light activity 15.3% and MVPA 2.9%), which was significantly greater than sedentary time during non-work time (68.9% p < 0.001). Office workers experienced significantly more sustained sedentary time (bouts >30 minutes) and significantly less brief duration (0–10 minutes) light intensity activity during work hours compared to non-work time (p < 0.001). Further, office workers had fewer breaks in sedentary time during work hours compared to non-work time (p < 0.001).

**Conclusions:**

Office work is characterised by sustained sedentary time and contributes significantly to overall sedentary exposure of office workers.

## Background

Sedentary behaviour is emerging as an important risk factor for poor health and for mortality [1-4]. Sedentary ‘activity’ is defined as any waking behaviour characterized by an energy expenditure ≤1.5 METs while sitting or reclining [5-7] Population research using self-report of sitting [2] or television viewing times [8] has found that as sitting or television viewing time increases there is an increased cardiometabolic risk, independent of moderate/vigorous physical activity (MVPA – activities requiring energy expenditures 3 or more times resting metabolic equivalents (METs) [7] such as brisk walking and running). Recently, high accelerometer determined sedentary time was related to metabolic and cardiovascular risk factors in healthy adults [3,9]. Furthermore, accelerometer determined prolonged or uninterrupted sedentary time has been found to be a risk factor for poor health independent of total sedentary time and MVPA [10]. Similarly, Healy et al. [11] found an association between light intensity physical activity (leisure, domestic or occupational activities requiring an energy expenditure of 1.6-3.0 METs [7] such as gentle walking) and plasma glucose levels in healthy adults, independent of MVPA; Camhi et al. [12] also found that light ‘lifestyle’ activity was associated with reduced odds of some cardiometabolic risk factors, again independent of MVPA.

With the evolution of the “technology age”, sedentary time is reported to be increasing [13-15]. This may be due in part to the shift towards reduced MVPA required in occupations traditionally requiring MVPA and the increasing percentage of workers employed in low activity occupations [16,17]. While sedentary workers may be less exposed to many of the hazards associated with more physically demanding occupations (e.g. manual labourers), sedentary workers may gain less of the beneficial MVPA and be exposed to more of the potentially detrimental prolonged and uninterrupted sedentary behaviour [5].

Whilst most research on physical activity has focused on non-occupational physical activity, some has examined physical activity at work and the relationship with leisure activity. Early studies using self-report measures found that workers in low activity occupations reported high levels of leisure time physical activity, suggesting that these workers were attempting to compensate for their lack of occupational activity [18,19]. In a recent review examining the relationship between occupation and leisure activity, Kirk and Rhodes[16] found mixed results with mainly self-report measures. However the majority of studies suggested that white collar workers tended to have greater leisure physical activity than blue collar workers. Further, in a recent study examining the MVPA of Scottish postal workers, it was found that there was no significant difference in the leisure time MVPA of walking postal workers compared to office based postal workers [20]. These studies focused on leisure time physical activity whereas other non-occupational physical activity related to transport and domestic duties are also thought to be important [5].

Due to the increasing prevalence of sedentary occupations and the potential contribution of work to sedentary risk, there is a growing interest in the occupational activity exposures of sedentary workers in terms of both the lack of MVPA and increase in sedentary exposure [17]. A recent review of occupational sitting and health risks [21] did not find sufficient evidence of a causal link between occupational sitting and poor health, partly because many studies did not adequately differentiate occupational and leisure time sitting. In one report for a health insurance company, based on accelerometer recordings, office workers were found to be sedentary for 76% of their working day [22]. While this report demonstrated that office workers have high sedentary exposure, the pattern of sedentary time was not fully explored. Ryan et al. [23], using an inclinometer-based device, examined university based office workers and their compliance with recommendations to take breaks from sitting every 20, 30 and 55 minutes during work hours. They found that 66% of the work day was spent sitting and taking frequent breaks in sitting was uncommon with 25% of the sitting time at work in bouts of 55 or more minutes. Whilst this study examined aspects of the pattern of sedentary time, it only examined sitting at work and did not measure light intensity activity or MVPA at work, nor did it explore sedentary time outside of work hours. Toomingas et al. [24], using inclinometers found that call centre operators were seated for an average of 75% of their working hours, with 9% of working hours spent sitting for periods of greater than 60 minutes. However, while this study did examine the pattern of sitting at work, it was not able to differentiate activity levels during non-sitting time.

Given the limited research that examines the pattern of exposure during work and outside work hours to all levels of objectively measured activity intensity, from sedentary time through light physical activity to MVPA, this study aimed to determine: 1) the proportions of time in sedentary, light and MVPA during work and non-work periods (work days versus non-work days, work time on a work day versus non work time on a work day, and work time on a work day versus all non work time); 2) the overall contribution of work sedentary time exposure to overall sedentary time exposure; 3) the pattern of sedentary, light and MVPA during work and non-work periods in terms of sustained sedentary periods, brief light activity periods and bouts of MVPA; 4) the relationships among measures of the pattern of sedentary time and physical activity; and 5) the relationships between work and non-work activity.

## Methods

### Design and subjects

A cross-sectional observational study was conducted with office workers (clerical and professional staff) from a large resource company in Perth, Australia. The company selected 12 work groups (20–40 employees per group) to attend a study recruitment meeting that was incorporated into a regular compulsory monthly meeting. At the end of the meeting, workers participating in office bound duties for 6 or more hours per day and working 4 or more days per week were asked to volunteer to participate in the study. Subjects were only excluded from participating if they were unable to wear an accelerometer due to disability or if they were confined to a wheelchair. 176 subjects completed physical activity surveys and 51 subjects from within this group also volunteered to wear an accelerometer for 7 days. One participant did not wear the accelerometer on non-work days and their data were excluded. All participants provided informed consent and ethics approval was obtained from the Human Research Ethics Committee of Curtin University, Perth WA (HR20/2007).

### Measurement of physical activity

The Actical (Phillips, Respironics) accelerometer is a small (2.8 × 2.7 × 1.0 cm), light (17 g) accelerometer that can be worn on the hip, wrist or ankle. It is described as “omnidirectional” as it detects movements in planes of movement other than the vertical. It has shown good technical reliability in laboratory studies [25] and validity as a measure of low energy expenditure behaviour in free-living conditions [26]. The Actical accelerometer has been used in a variety of population groups [27-29] and recently to evaluate sitting time of office workers [30].

### Procedure

Participants were asked to wear the accelerometer for 7 days [31,32]. The accelerometer was set to record data using a 60 second epoch [33]. It was attached to an elastic belt and worn over their right hip [34] for all waking hours. Activities, accelerometer wear time and the reason why the accelerometer was removed (e.g. bathing, contact sport), waking hours and work hours (from the time seated at a desk/workstation until leaving the office) were recorded in a simple diary. Sedentary time, light and MVPA during work time and non-work hours (wear time before and after work on work days and wear time on non-work days) were then examined and compared. Non-work physical activity (activities that occurred outside of work hours) included leisure activities such as sport and brisk walking but also incorporated active transport and domestic chores.

### Data processing

Actical raw data were downloaded using the manufacturer’s software and then raw activity count data were processed using a custom LabVIEW program (LabVIEW 8.6.1 National Instruments, Texas, USA). The LabVIEW program enabled detailed simultaneous analysis of the pattern of activity intensity and duration to be studied using Exposure Variance Analysis [35]. Activity intensity categories of sedentary, light, moderate and vigorous were determined from counts per minute. As counts are arbitrary and device specific [36], intensity category cut points (sedentary < 91 counts, light 91- < 1767 counts, moderate 1767- < 5182 counts and vigorous >5182) were based on those widely used for Actigraph accelerometers (sedentary < 100 counts, light 100- < 1951 counts, moderate 1951- < 5275 counts and vigorous >5275 [37] but translated to Actical using an equation based on a study collecting biological data simultaneously from Actigraph and Actical accelerometers [38]. Duration was characterised as bouts within the same intensity lasting 0- < 5mins, 5- < 10mins, 10- < 30mins, 30- <60 mins and 60+ mins to match other research and recommendations [38-40]. Non-wear during waking hours was firstly determined from diary entries and then during the data processing. Periods greater than 120 minutes with counts of zero were considered non-wear time, rather than periods of greater than 60 minute as pilot testing observations showed some office workers were sustaining sedentary time for greater than 60 minute bouts. A break in sedentary time was defined as accelerometer counts above 91 counts/min (Actical translated break cutpoint) for greater than one minute during sedentary periods [10]. While minimum wear time of 600 minutes/day has been used in some studies [10,31], minimal wear time was set at 500 minutes/day [41,42] to limit participant burden and to maximise the data that could be used in analysis. Only 12 of the 359 days included had wear time between 500–600 minutes. Days with less than 500 minutes were automatically discarded and not included in the data processing. Participants were required to wear the accelerometer for a minimum of 3 work days and 1 non-work day to be included in data processing [43,44]. Measures of the pattern of activity extracted from the Exposure Variation Analysis and of particular interest for this paper were sustained (>30mins) periods of sedentary time, brief (0-5mins and 5-10mins) periods of light activity and bouts (>10mins) of MVPA.

### Statistical analysis

Paired t-tests were used to compare time in activity levels between work and non-work days and between work hours on work days and non-work periods. Correlations between activity levels at work and non-work periods were performed using Pearson’s correlations. All calculations were made using the percentage of wear time for each time period. All analysis was done using PASW Statistics 18 with a critical alpha level of 0.05.

## Results

50 participants (58% men) aged between 22 and 59 years (mean ± SD, 36.4 ± 8.6 years) with a BMI of 24.7 ± 4.1 kg/m^2^ completed the study and wore an accelerometer for an average of 7.0 ± 0.9 days (4.6 ± 0.5 work days and 2.4 ± 0.9 non-work days). A total of 231 valid work days and 121 valid non-work days were included in analysis. The average accelerometer wear time for work days was 14.9 hours (892.5 ± 65.5 mins) which was significantly greater than the 13.7 hours wear time for non-work days (820.6 ± 85.5 mins, t = 6.0, df =49, p < 0.001). Wear time for work hours was 8.9 hours (535.2 ± 46.2 mins) which was 60.0% on the total work day wear time.

### Sedentary time, light and moderate/vigorous physical activity on work and non-work days

**Figure 1:**
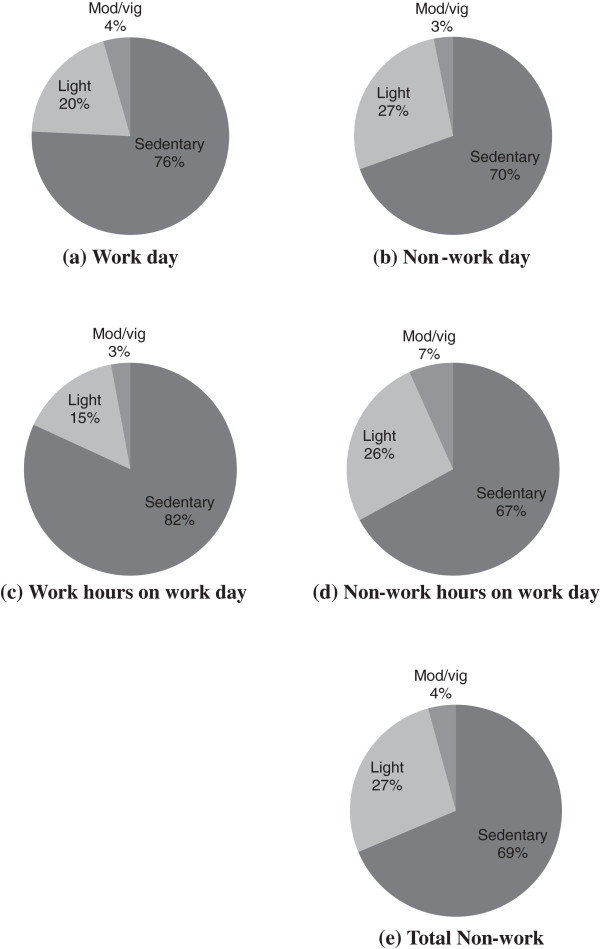
Proportion of time in sedentary, light, moderate and vigorous activity on a work day (a), non-work day (b), work hours on a work day (c), non-work hours on a work day (d) and total non-work time over a whole week.

Sedentary time on work days of 11.3 hours per day ([676.0 ± 58.7 mins] 75.9% wear time) was proportionally greater than the 9.3 hours per day on non-work days ([570.5 ± 88.0 mins], 69.7% wear time, t = 6.2, df = 49, p < 0.001). Light activity on work days of 3.0 hours ([176.9 ± 52.6 mins] 19.7% wear time) was proportionally less than the 3.7 hours on non-work days ([224.4 ± 78.3 mins] 27.2% wear time, t = −7.8, df = 49, p < 0.001). MVPA on work days of 39.5 ± 18.7 mins (4.4% wear time) was proportionally greater than the 25.7 ± 25.7 mins on non-work days (3.1% wear time, t = 3.3, df = 49, p = 0.002). Figures 1(a) and 1(b) illustrate these differences. 78% of all participants had proportionally more sedentary time on work days compared to non-work days and 84% of participants had proportionally less light activity on work days compared to non-work days.

### Sedentary time, light and moderate/vigorous physical activity at work and during non-work hours on a work day

On work days, sedentary time was proportionally greater during work hours compared to non-work hours (work hours [438.3 ± 51.5 mins] 81.8% wear time, non-work hours on work day [237.7 ± 50.7 mins] 67.0% wear time, t = 12.7, df = 49, p < 0.001) and there was proportionally less light intensity activity during work hours on work days compared to non-work hours on work days (work hours [81.6 ± 25.6mins] 15.3% wear time, non-work hours on work day [95.3 ± 39.2 mins] 26.2% wear time, t = −9.6, df = 49, p < 0.001). MVPA of 15.6 ± 9.4 mins (2.9% wear time) during work hours on a work day was proportionally less than the 24.0 ± 14.2 mins (6.8% wear time) during non-work hours on work day (t = −6.9, df = 49, p < 0.001)(see Figures 1(c) and 1(d)).

### Sedentary time, light and moderate/vigorous physical activity at work and during all non-work hours over a whole week

A similar pattern of results was found when comparing work hours on work days with all non-work hours over a whole week. The total non-work hours includes the time before and after work and all non-work days, equating to 56% of total wear time. Sedentary time was proportionally greater during work hours (81.8%) compared to total non-work time ([808.2 ± 115.4 mins], 68.9% wear time, t = 10.8, df = 49, p < 0.001). Light intensity activity was proportionally less during work hours (15.3%) compared to total non-work time ([319.7 ± 109.0 mins] 26.9% wear time, t = −10.5, df = 49, p < 0.001) and MVPA was proportionally less during work hours (2.9%) compared to total non-work time ([49.7 ± 33.7 mins], 4.2% wear time, t = −3.0, df = 49, p < 0.050) (See Figures 1(c) and 1(e)).

### Overall contribution of occupational sedentary time to total sedentary time

In terms of total weekly sedentary time, work time contributed 36.5 hours (48.5% of total sedentary time) with all non-work time contributing 38.7 hours (51.5%).

### Pattern of sedentary time, light activity and MVPA

Table 1 presents the selected Exposure Variation Analysis variables to represent key aspects of the pattern of sedentary, light and MVPA time for work and non-work periods as well as the number of breaks in sedentary time.

**Table 1:** Selected Exposure Variance Analysis variables demonstrating patterns of sedentary time, light activity and MVPA during work and non-work periods

	**Type of day**		**Wear time on work day**		**Total non-work time**
	**Work day**	**Non-work day**	**Work hours on work day**	**Non-work hours on work day**	
Sustained sedentary time >30 mins (%wear time ± SD)	34.1 ± 11.6 ^#^	26.9 ±11.1	40.8 ±16.6 ^	22.8 ± 10.9	25.8 ± 9.6
Light activity bouts 0–5 mins (%wear time ± SD)	13.4 ± 2.2 ^#^	14.6 ± 2.8	12.2 ± 3.1 ^	15.4 ±2.2	14.8 ± 2.3
Light activity bouts 5–10 mins (%wear time ± SD)	4.1 ± 2.1 ^#^	7.5 ± 3.7	2.6 ± 1.7 ^	6.5 ± 3.4	7.2 ± 3.3
MVPA bouts > 10 mins (% wear time ± SD)	1.2 ± 1.6	1.1 ± 1.9	0.5 ± 1.0^	2.2 ± 3.2	1.4 ± 2.0
Breaks in sedentary time (breaks/sed hour ± SD)	6.0 ± 1.4 ^§^	9.2 ± 9.8	5.1 ± 1.7 ^	7.9 ± 2.1	8.0 ± 2.6

^§^ Significant difference between work and non-work day (p < 0.05).

^#^ Significant difference between work and non-work day (p < 0.001).

^ Significant difference between work hours on a work day and non-work hours on a work day and between work hours on a work day and total non-work time (p < 0.001).

Sustained sedentary time (bouts >30 mins) was proportionally greater on work days compared to non-work days, and also during work hours on work days compared to non-work hours on work days and total non-work time over a whole week. Weekly work time sustained sedentary time (bouts > 30 mins) was 18.2 hours/week making work time account for 56.7% of total weekly sustained sedentary time (32.1 hours/week). Prolonged sustained sedentary bouts (sedentary bouts > 60 mins) accounted for 12.7 hours over a whole week.

Brief periods of light intensity activity were proportionally less on work days compared to non-work days, and also during work hours on work days compared to non-work hours on work days and total non-work time. The majority of MVPA occurred in bouts of < 10 mins, with only 93.9 minutes per week of MVPA accumulated in bouts of 10 minutes or greater. 14% of participants had > 150 minutes per week of moderate intensity activity in bouts of 10 or more minutes, additionally 14% of participants had >60 minutes per week of vigorous intensity activity in bouts of 10 or more minutes. Bouts of MVPA >10mins were proportionally less during work hours on work days compared to non-work hours on work days and total non-work time. Office workers also had significantly less breaks in sedentary time on work days and during work hours on work days.

**Figure 2:**
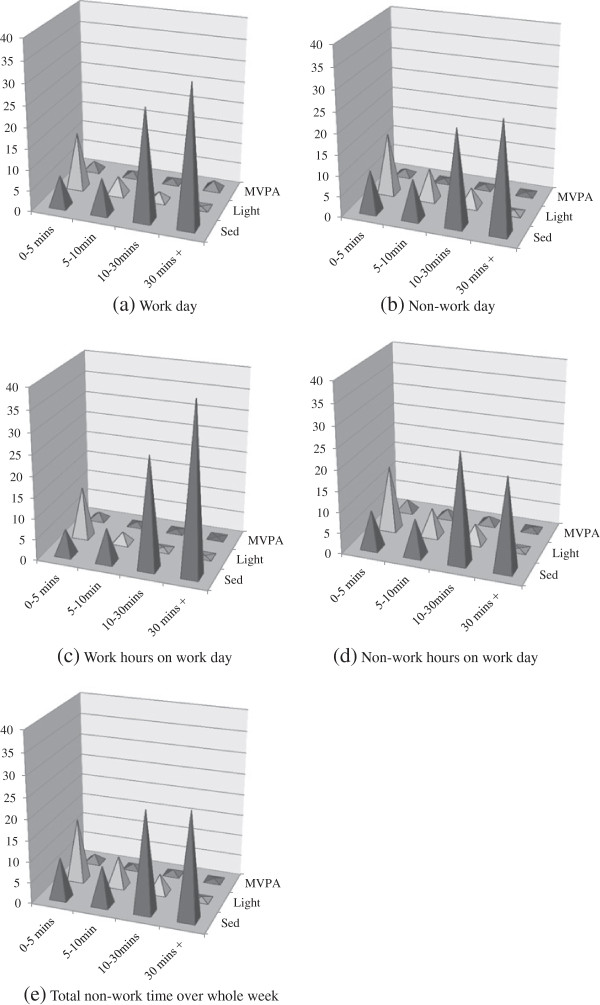
Exposure Variance Analysis showing proportion of wear time in sedentary, light and MVPA in bouts of 0- < 5, 5- < 10, 10- < 30 and 30+ minutes on a work day (a), non-work day (b), work hours on a work day (c), non-work hours on a work day (d) and total non-work time over a whole week (e).

Comparison of both the intensity of physical activity and the duration of sustained activity during work and non-work periods is shown in Figure 2. The taller columns for sustained sedentary time and the shorter columns for brief bouts of light activity during work hours on a work day 2(c) highlight the differences in exposure pattern between work and non-work periods. MVPA columns are all very short as MVPA only accounted for 4.1% (252.4 mins) of total wear time.

### Relationship between sedentary and physical activity variables

Sustained sedentary time greater than 30mins was strongly negatively associated with the number of breaks in sedentary time (on work days (r = −0.85, p < 0.010), on non-work days (r = −0.74, p < 0.010), during work hours on work days (r = −0.93, p < 0.010), and in total non-work time (r = −0.74, p < 0.010)). There was a strong negative relationship between sedentary time at work and light activity at work (r = −0.96, p < 0.010). There was also a significant negative relationship between sedentary time at work and MVPA at work (r = −0.65, p < 0.010).

### Relationship between work and non-work time activity

The proportion of sedentary time at work was moderately associated with the proportion of sedentary time during non-work hours (r = 0.38, p = 0 < 0.010) and was moderately *negatively* associated with the proportion of light intensity activity during non-work hours (r = −0.41, p < 0.010). The proportion of sedentary time at work was not significantly associated with the proportion of non-work MVPA (r = −0.010, p = 0.925). Further, there was no significant association between the proportion of MVPA at work and the proportion of MVPA during non-work periods (r = 0.17, p = 0.234).

## Discussion

This study specifically examined for the first time the accumulation and pattern of sedentary time, light activity and MVPA of office workers during work time and non-work time (non-work hours on a work day and non-work days) and found work time contributed significantly to overall exposure to sedentary time and sustained sedentary time.

Office workers in this study were sedentary for 81.8% of work hours on work days, similar to the only prior accelerometer determined comparison data of Thorpe et al. (2012) (office workers were sedentary for 75.8% of work hours on a work day and call centre workers were sedentary for 82.0% of work hours on a work day). Sitting time of office workers was less (66% of working hours) in a study by Ryan et al. [23] using activPAL, though similar (75% of work hours) in a study using inclinometers reported by Toomingas et al. [24]. Differences found in sedentary time may be due to the variation in the nature of work performed (eg clerical/administrative, call centre or university academic) and work practices (organisational culture regarding breaks and productivity or output) as well as differences between devices used. While inclinometer based devices, such as activPAL, differentiate between postures (lying/sitting/standing) [45], movement based devices, such as accelerometers differentiate between the intensity of movement and have the advantage of providing simultaneous detail about sedentary, light and MVPA [44].

Regardless of measurement device, it is clear that office workers can be sedentary for a very high proportion of their work hours, and this study found that sedentary time during work hours accounted for nearly half (48.5%) of their total weekly sedentary time. This study has therefore clearly established that work is an important contributor to overall weekly sedentary exposure for office workers and thus to their associated health risks [3,9]. Future research should investigate how to reduce work sedentary exposure.

Further, the study findings showed only a small proportion of work hours were spent in light activity and for the first time showed that there was a strong reciprocal relationship between sedentary time and light activity during work hours. Participation in light activity is thought to be beneficial [46] and thus health promotion interventions, particularly in workplace settings, should target this interplay between light and sedentary activity with the aim of replacing sedentary time with light activity. Future research should attempt to determine the amount of light activity at work necessary to provide a health benefit. While it may not be feasibly in all office environments, providing the opportunity to spend some time during work hours standing by use of a sit/stand work station [24,47], could encourage light intensity activity with minimal impact on work productivity. Further, time away from a desk does not necessarily need to be non-productive - workplaces could encourage incidental office activity such as ‘active e-mails’ or walking meetings. Future workplace interventions to reduce sedentary behaviour should therefore consider the effect on work productivity and attempt to measure if programmes have a positive or negative impact on productivity.

Office workers spent the majority of their time, both at work (97.1%) and during non-work hours (95.7%), in either sedentary or light activity with MVPA only accounting for a very small proportion of wear time. These findings were consistent amongst participants with the majority of participants more sedentary (78%) and having less light activity (84%) on work days compared to non-work days. Most health promotion interventions have focused on encouraging sufficient MVPA to promote health benefits and prevent many chronic diseases (eg “findthirty” campaign that encourages 30 minutes of daily activity for good health [48]). Given that the vast majority of the week is spent in sedentary or light activity, and the emerging evidence of the health impacts of sedentary and light activity [3,9,11] public health campaigns should consider implementing programmes to modify sedentary and light activities.

Office workers in this study experienced more prolonged, uninterrupted sedentary time, fewer breaks in sedentary time and fewer brief bouts of light intensity activity during work hours compared to non-work periods. Given the evidence suggesting fewer breaks interrupting sedentary time are associated with greater health risk [10], the current study demonstrates that the pattern of sedentary and activity exposure of office work increases health risks for office workers. Further, this study found that office workers were sedentary in bouts of greater than 60 minutes (and less than 120 minutes) for over 12 hours a week. Studies that estimate non-wear time by the use a 60 minute cut point of continuous activity counts of zero, could potentially be misclassifying prolonged sustained sedentary time as non-wear time. Office work commonly involves sitting at a desk interacting with computer and paper based information. The work itself thus provides little of the potentially beneficial light activity or MVPA. Whilst workplace interventions to date have tended to focus on incorporating activity between productive work [49,50] attempts to change the nature of productive work to break up sustained sedentary time with light activity are being developed (sit-stand desks,[24,47], walking/cycling desks [51,52]). These approaches may be more acceptable to organisations as there is only minimal impact on work productivity [52].

Even though there was a strong negative relationship between sustained sedentary time and breaks in sedentary time, it is not possible to determine whether they are capturing the same risk construct: prolonged sedentary time may have unique and different physiological consequences when compared to engagement in active breaks [46,53]. This issue is analogous to the link between musculoskeletal disorders and sustained low level muscle contractions [54]. In muscle physiology studies examining breaking up sustained low level muscle contractions, changes in muscle activation patterns are seen following breaks composed of total rest as well as following breaks composed of greater muscle activity [55,56]. Thus it may be that sustained sedentary time results in a detrimental physiological state but breaks of a sufficiently active nature may result in a beneficial physiological state. Recent experimental evidence has shown even brief breaks of light intensity activity can improve glucose metabolism [57]. Thus the current findings suggest that research should be conducted examining the physiology of both sustained sedentary and activity breaks necessary to understand the underlying mechanisms of effect, and findings from these used to develop public health messages around avoiding sustained sedentary exposure and/or seeking out active breaks.

The relationship between work and non-work activity is likely to be complex [16] and influenced by a variety of factors such as job, family and individual characteristics. The present study found that high sedentary time at work was not associated with ‘compensatory’ MVPA outside of work hours. However, such attempts to ‘compensate’ may be in vain as recent evidence has shown that sedentary time and breaks in sedentary time are independently associated with health risks [3,10], that is they can not be compensated for by increased MVPA. There were higher levels of MVPA on work days compared to non-work days. Examining the work time on a work day results, showed that the higher levels of MVPA were accumulated during non-work time on work days. Whilst we did not collect data on what activities contributed to these higher levels of MVPA, both active transport to and from work and/or participation in leisure time activities and sport, are likely to have been important contributors to MVPA exposure. Of interest were the findings that 26% of participants achieved physical activity recommendations for MVPA in bouts of >10 minutes [58], which is a higher proportion that has been found in large population research [31,59]. These results may be a product of living in a city with a climate and culture that is very conducive to participation in physical activity and also, the participants in general were well educated and worked in a corporate environment that encouraged and promoted physical activity.

Interestingly, this study found that people that were sedentary at work tended to also be more sedentary and have less light activity outside of work hours. Thus sedentary office workers may be at greater risk of poor health associated with sedentary behaviour due to sedentary exposure both at work and outside of work. Sedentary workers may have self-selected a sedentary occupation and may be at particular risk due to some predisposition to be sedentary. Therefore, workplace interventions that solely target work practices may have limited benefit as interventions may need to modify individual attitudes and beliefs. However, there is also the possibility that workplace interventions aimed at reducing sedentary time at work could result in changes to sedentary behaviours in non-work hours, as workplace interventions for smoking, alcohol, stress and weight loss have successfully reduced these adverse health behaviours during non work hours [60,61] and therefore reducing sedentary time at work could have a magnified impact on reducing overall weekly sedentary exposure.

A strength of this study is that it is the first to comprehensively explore the total accumulation and pattern of sedentary time, light activity and MVPA of office workers during work time and outside of work time. The study analysed work hours as distinct from work days [62] which allowed for separate examination of activity levels during non-work time on a work day. The study also used an objective measure of sedentary time, light activity and MVPA rather than self report. Limitations of the study include the moderate sized sample from a single organisation in one city and use of only a movement intensity device without the addition of a posture recording device. There may have been a selection bias to more active participants as those who volunteered may be more likely to be active which could have resulted in an underestimate of sedentary time in the cohort. Accelerometer wear time was less on non-work days, with later start times observed. In the future a comprehensive examination of sedentary time for other occupational groups with 24 hour monitoring would be beneficial.

## Conclusion

Office work contributes significantly to overall sedentary exposure and therefore the associated health risks of sedentary behaviour. Furthermore, compared to non-work periods, occupational sedentary time of office workers was significantly more prolonged with fewer breaks. Although office work has traditionally been considered a ‘low risk’ occupation in terms of chronic health outcomes, it may in fact increase the risk of mortality and cardiometabolic disorders due to overall accumulated sedentary time and especially sustained sedentary time at work. Given the evidence for a health impact of sedentary and light activity, work based activity interventions should therefore target reducing total sedentary time and also emphasise the importance of interrupting sedentary time and provide an opportunity to participate in light intensity activity.

## Competing interests

The authors declare that they have no competing interests.

## Authors’ contributions

SP and LS conceived and designed this study, SP collected and analysed the data with the assistance and supervision of LS. SP and LS drafted the manuscript. All authors read and approved the final manuscript.

## Pre-publication history

The pre-publication history for this paper can be accessed here:

http://www.biomedcentral.com/1471-2458/13/296/prepub
